# Supplementary material for: Correlated states in doubly-aligned hBN/graphene/hBN heterostructures
Source: Nat Commun. 2021 Dec 10;12:7196. doi: 10.1038/s41467-021-27514-y (PMC8664858; doi:10.1038/s41467-021-27514-y)
Supplement: Supplementary file 1 — Supplementary Information [file 41467_2021_27514_MOESM1_ESM.pdf]

Supplementary Information for

"Correlated states in doubly-aligned hBN/graphene/hBN heterostructures"

Xingdan Sun, Shihao Zhang, Zhiyong Liu, *et al.*

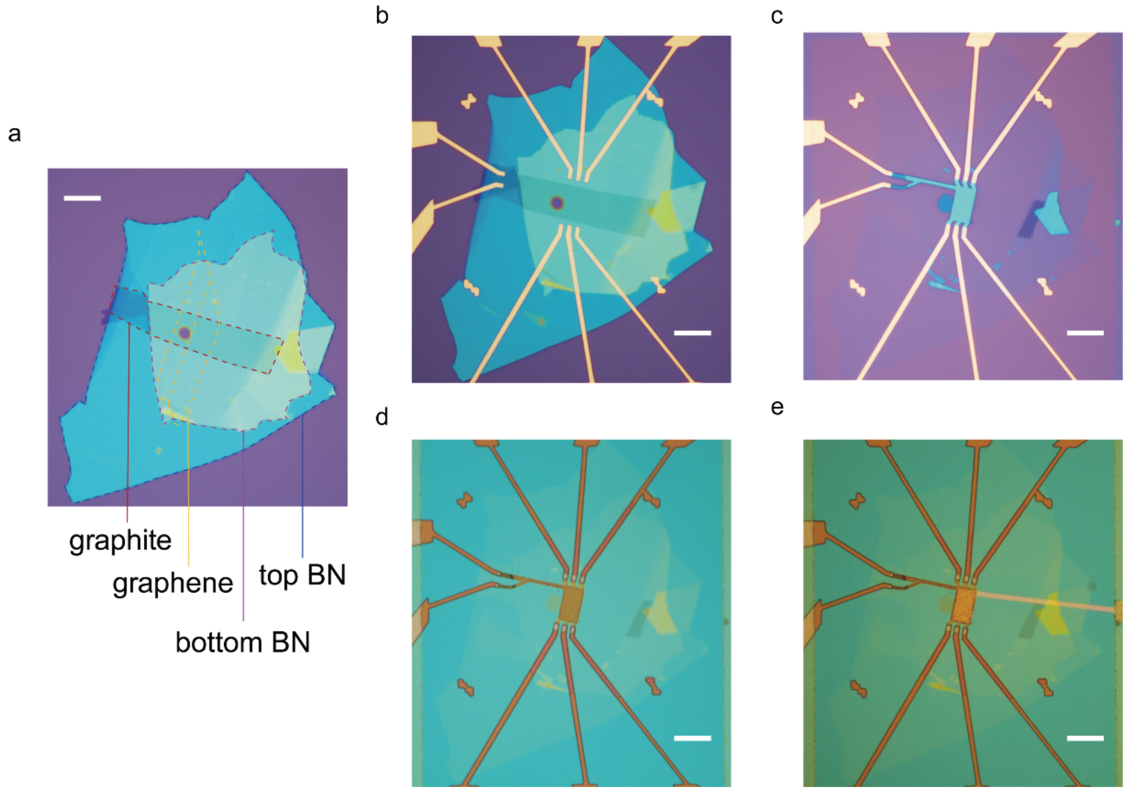

Supplementary Figure 1. **Optical microscopy images on fabrication process of sample S2.** (a). h-BN/graphene/h-BN/graphite vertical heterostructure fabricated using the dry transfer method. Scale bar is 10 μm. [(b)-(e) share this scale]. (b). Ti/Au electrodes deposition with patterned PMMA mask. (c). Patterning of a Hall bar. (d). The deposition of HfO_2 by atomic layer deposition (ALD) as top-gate dielectric. (e). Deposition of gold top gate.

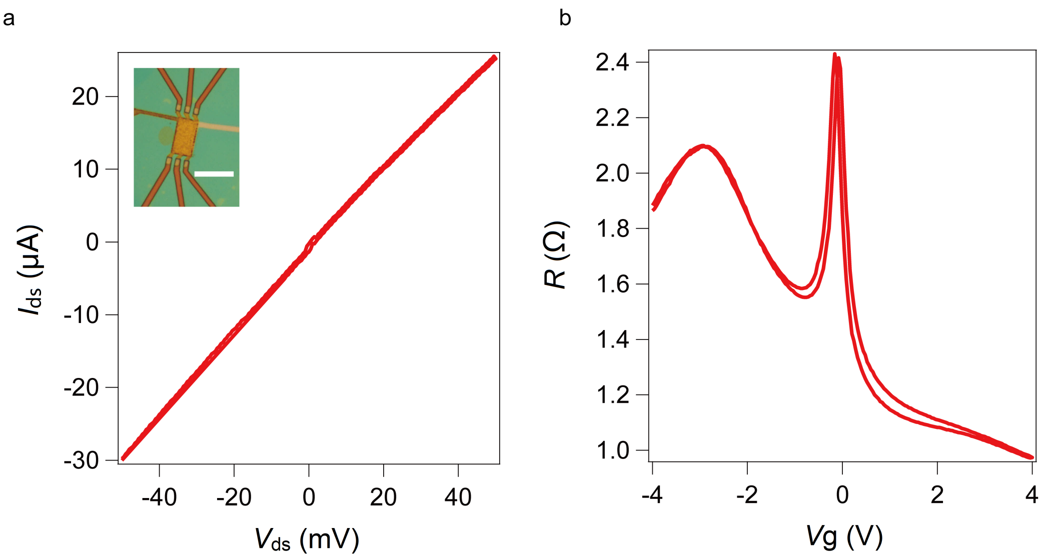

Supplementary Figure 2. **Electrical characterization of sample S2.** (a). $I-V$ characteristics measured at zero gate voltage at room temperature. The inset shows an optical image of a typical device, scale bar is 10 μm. (b). Two-terminal resistance versus gate voltage at room temperature, and the emergence of satellites of the charge neutral point (CNP) indicates a small angle near zero between graphene and h-BN.

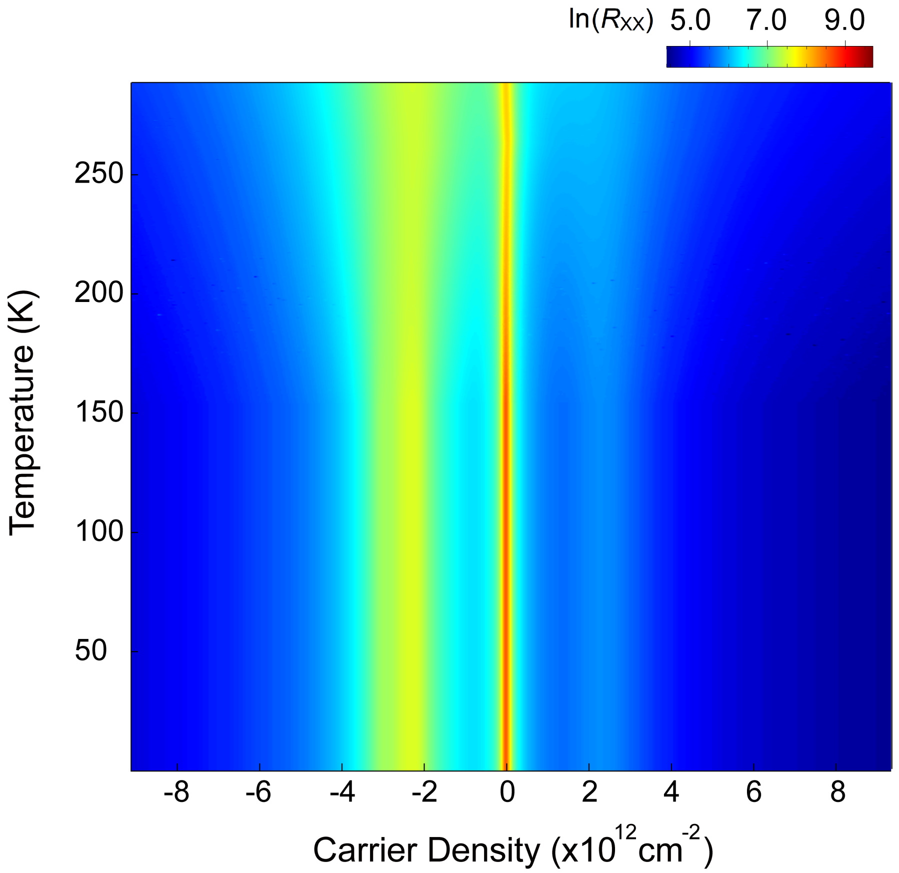

Supplementary Figure 3. **Temperature dependence of the resistance for sample S2.** The mapping of the longitudinal resistance, R_{xx}, is shown as a function of temperature and carrier density, and the temperature range is 0.085-300 K.

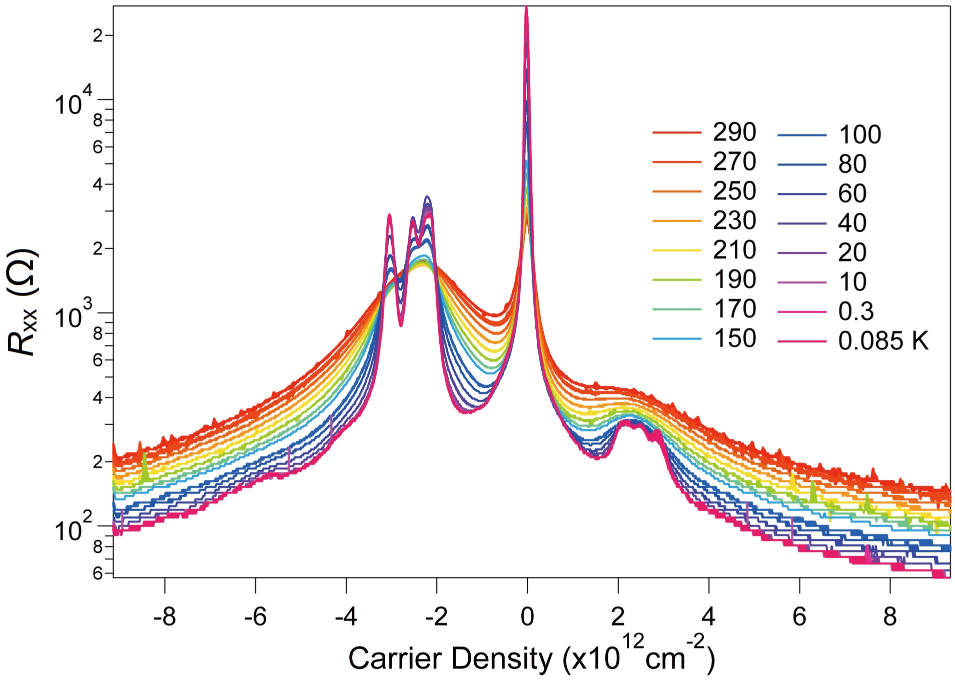

Supplementary Figure 4. **Temperature dependence of the resistance for sample S2.** R_{xx} as a function of carrier density at different temperatures, extracted from Supplementary Figure 3.

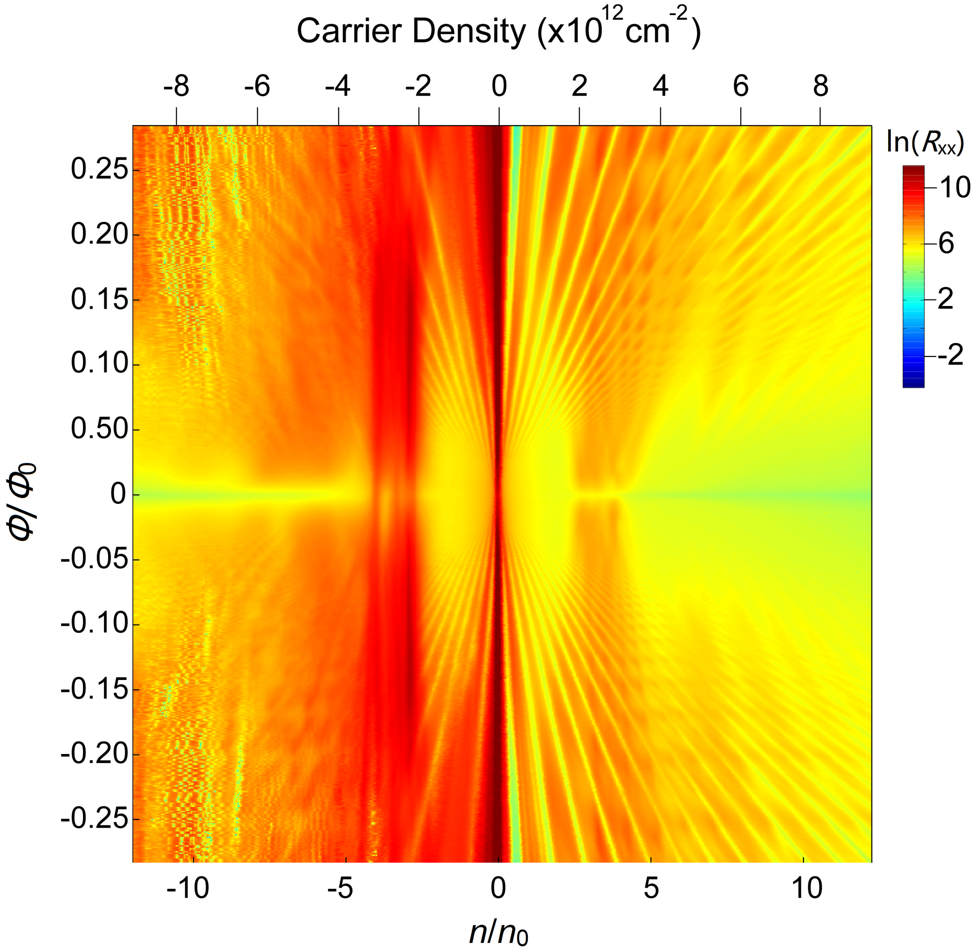

Supplementary Figure 5. **Hofstadter butterfly for sample S2.** Landau fan diagram for device S2. R_{xx} is plotted versus magnetic field on the vertical axis and versus gate bias on the horizontal axis. In the diagram showing R_{xx}, the axes are scaled by the size of the moiré unit cell to give ϕ/ϕ_0 on the vertical axis and n/n_0 on the horizontal axis. The carrier density is shown on the top axis. Measurements are acquired at 8.5 mK. $n/n_0 = 4$ corresponds to full filling of the moiré unit cell.

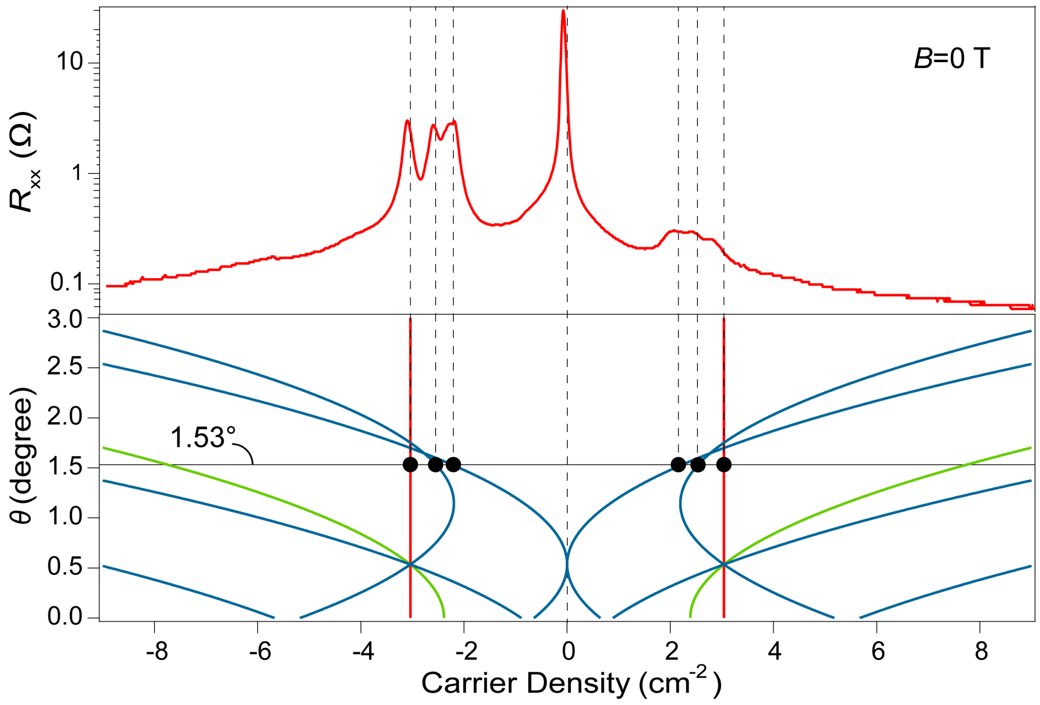

Supplementary Figure 6. **Locating resistive peaks in sample S2.** The analysis of twist angle of sample S2 from the phenomenological formula in Eq. (2) in the main text. Field effect curve in the upper panel was obtained at 50 mK.

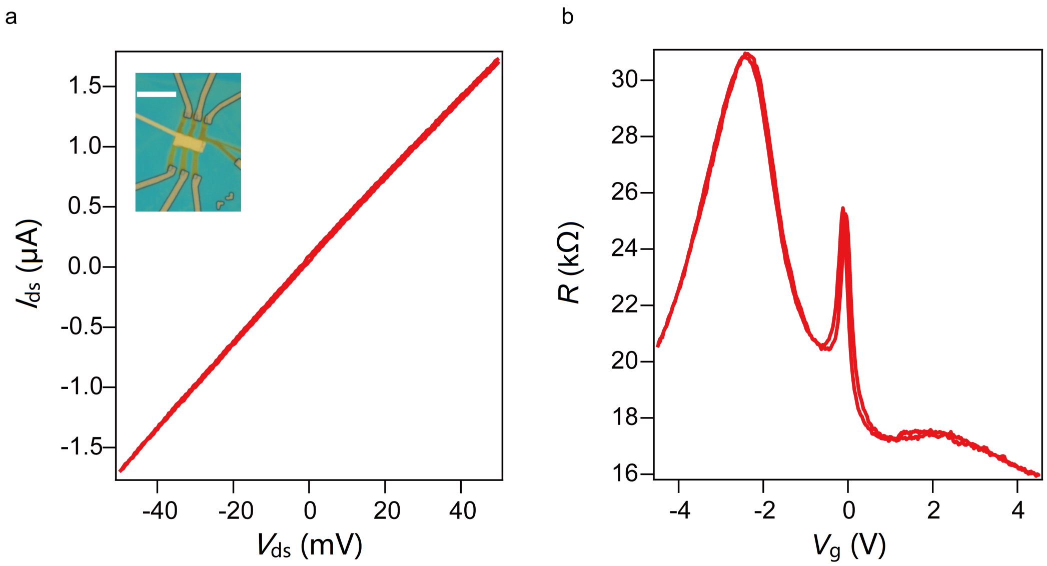

Supplementary Figure 7. **Electrical characterization of sample S12.** (a). *I*-*V* characteristics measured at zero gate-voltage configurations at room temperature. The inset shows an optical image of a typical device, scale bar is 10 μm. (b). Two-terminal resistance versus gate voltage at room temperature.

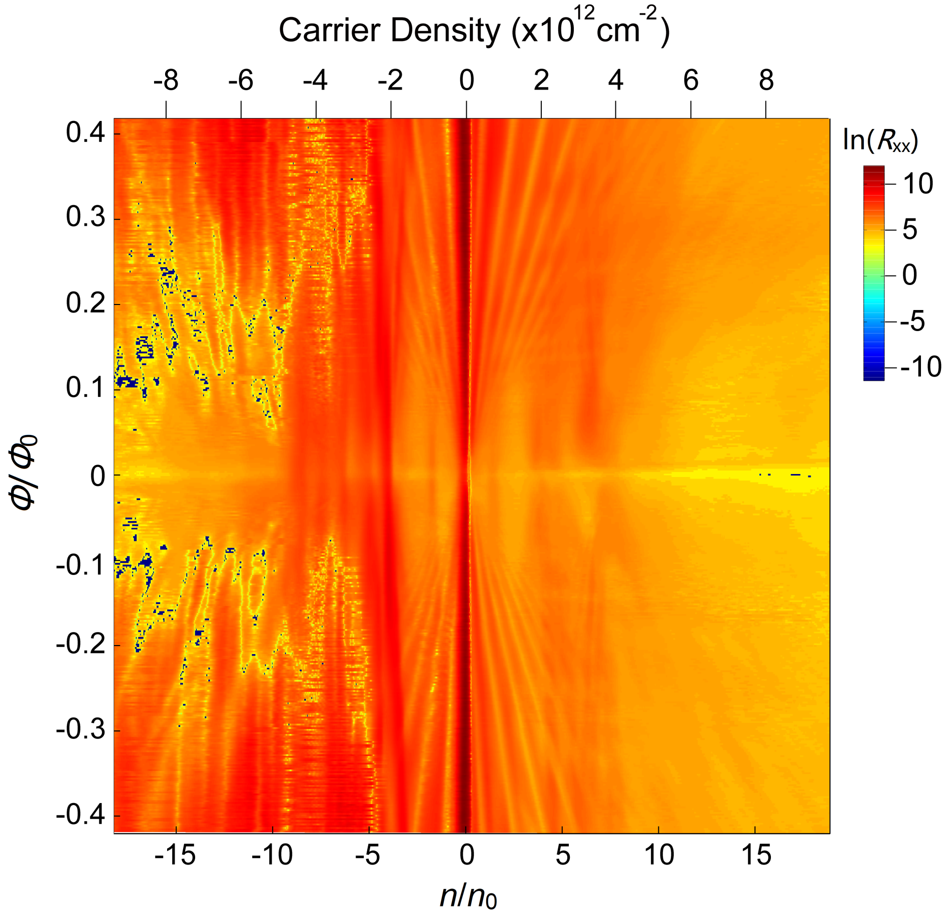

Supplementary Figure 8. **Hofstadter butterfly for sample S12.** Landau fan diagram for device S12 plotted in ϕ/ϕ_0 and n/n_0. Data measured at 50 mK.

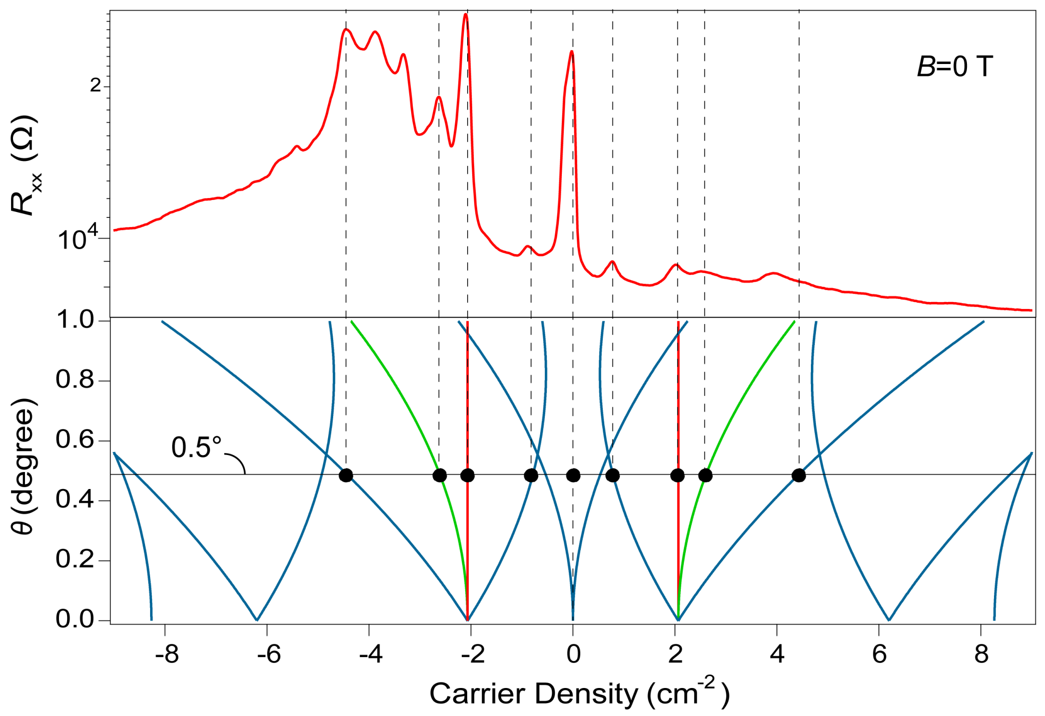

Supplementary Figure 9. **Locating resistive peaks in sample S12.** The analysis of twist angle of sample S12 from the phenomenological formula in Eq. (2) in the main text. Field effect curve in the upper panel was obtained at 50 mK.

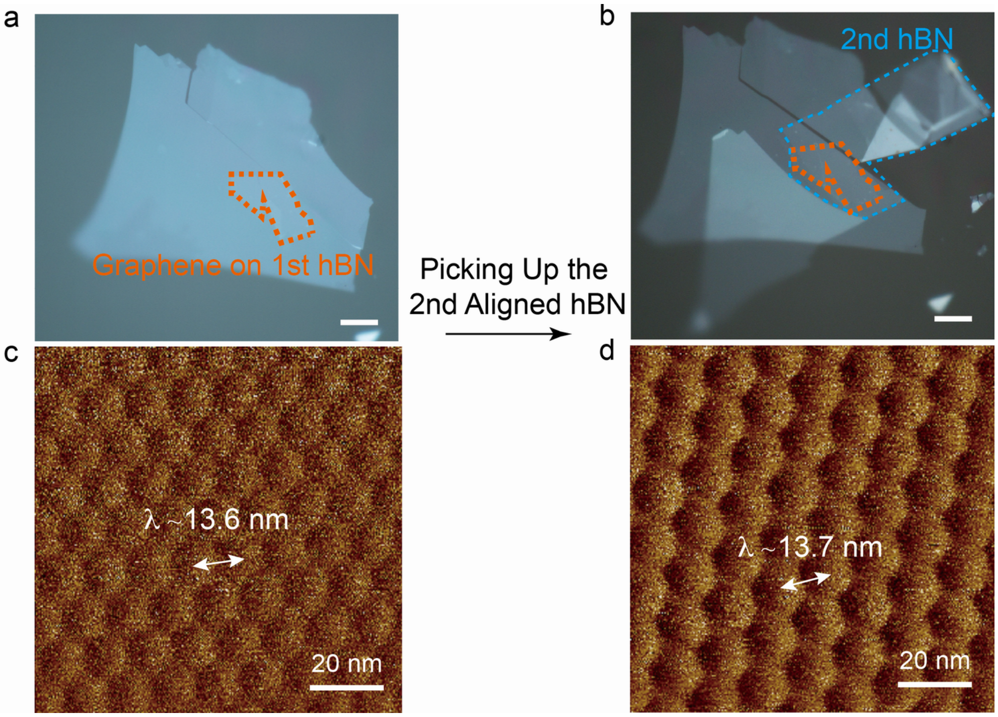

Supplementary Figure 10. **Direct verification of the dual moiré length-scale.** Moiré wavelength of about $\lambda \sim$13.6 nm was seen after each pick-up step in the sample fabrication process. (a)-(b) and (c)-(d) are optical images and atomic force microscopy (AFM) images with a friction force mode, respectively. An OXFORD qp-BioAC-20 tip was used for the AFM friction characterization. Scale bars in optical images in (a)-(b) are 10 μm.

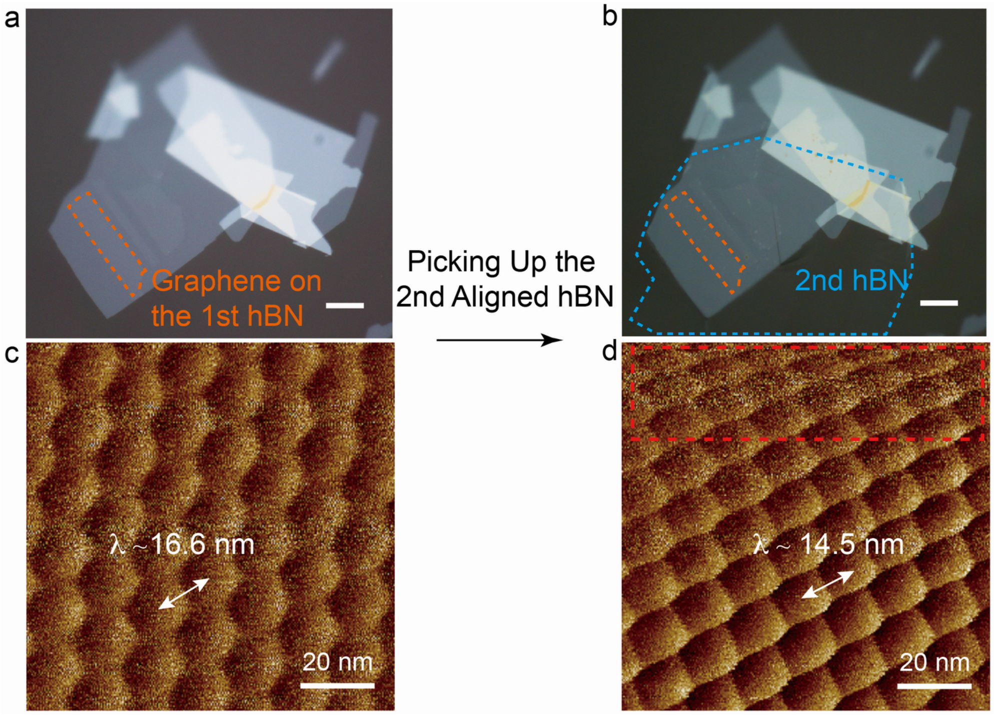

Supplementary Figure 11. **AFM scan of dual moiré length-scale obtained in another Sample.** $\lambda \sim$ 16.6 nm (indicating an existing strain, which is in agreement with other reports [10]) was found in the first pick up, while $\lambda \sim$ 14.5 nm was seen for the second pick up. Inhomogeneous regions can be seen in the red dashed box, indicating that the doubly aligned area can be rather local, and this is why the device yield is very low (less than 1/20). Scale bars in optical images in (a)-(b) are 10 μm. Clear inhomogeneities in the moiré superlattices can be seen in the red dashed box area, indicating that the well aligned area can sometimes be rather local, with micronmeter sizes or smaller. This kind of inhomogeneities in moiré superlattice is also reported elsewhere.[11]

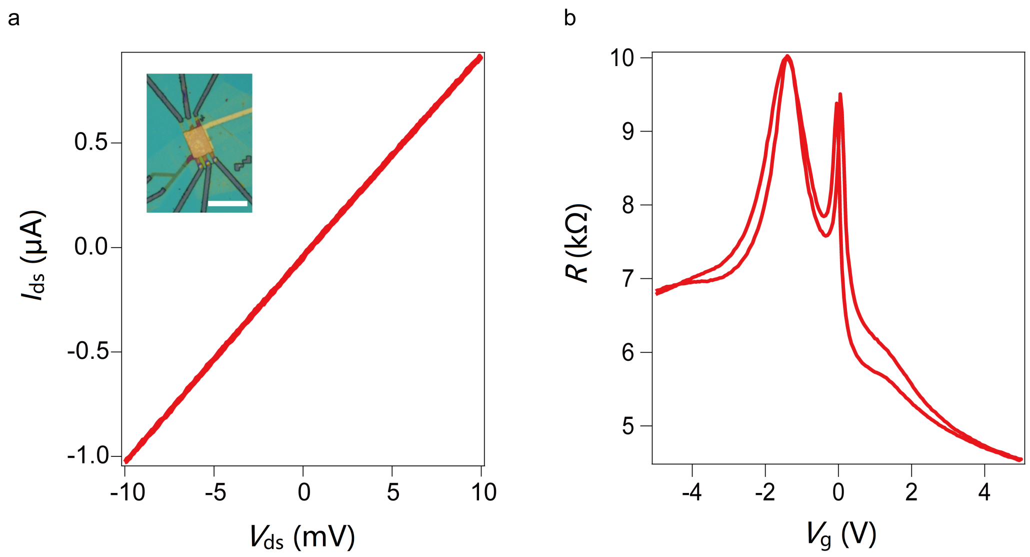

Supplementary Figure 12. **Electrical characterization of sample S7.** (a). *I*-*V* characteristics measured at zero gate-voltage configurations at room temperature. The inset shows an optical image of a typical device, scale bar is 10 μm. (b). Two-terminal resistance versus gate voltage at room temperature. All relative analysis of sample S7 are given in the main text.

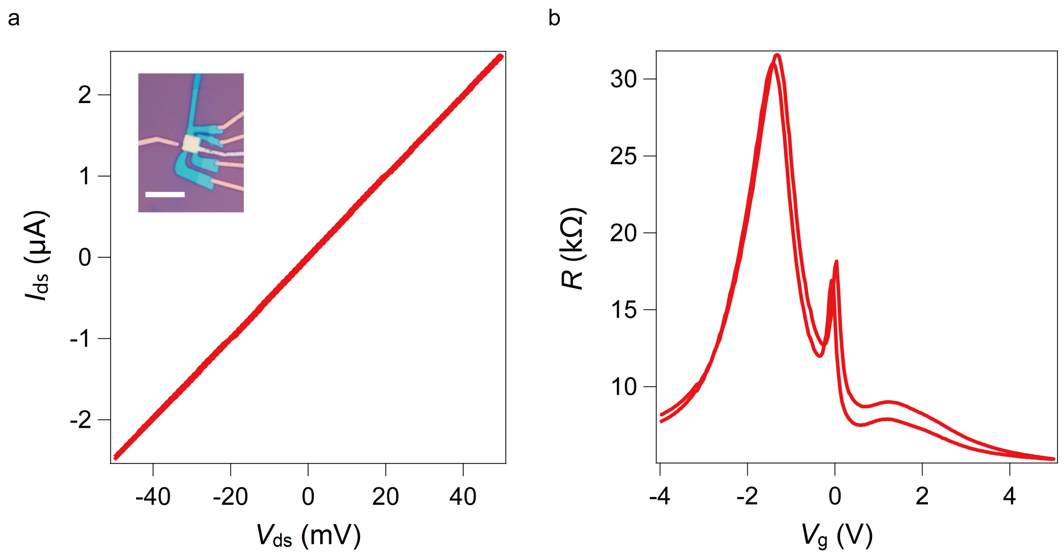

Supplementary Figure 13. **Electrical characterization of sample S4.** (a). *I*-*V* characteristics measured at zero gate-voltage configurations at room temperature. The inset shows an optical image of a typical device, scale bar is 10 μm. (b). Two-terminal resistance versus gate voltage at room temperature. Sample S4 has only two electrodes working, and we could only perform two probe measurements for this sample.

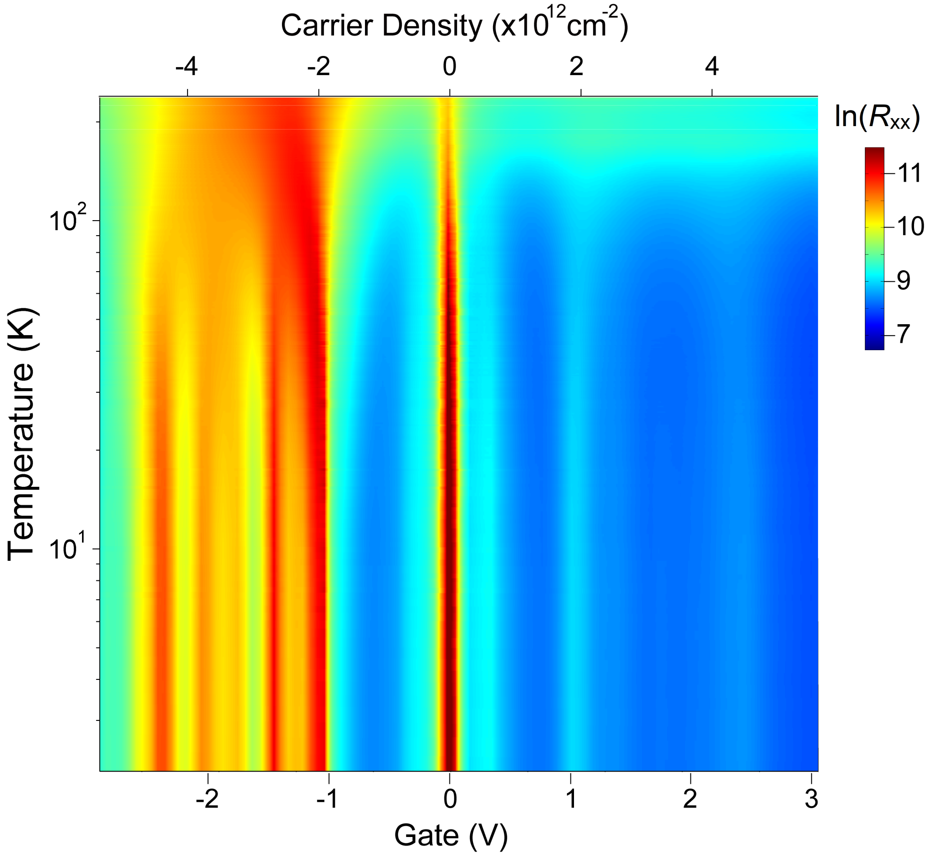

Supplementary Figure 14. **Temperature dependence of the resistance for sample S4.** Color map of the sample resistance as a function of gate voltage and temperature, and the carrier density is shown on the top axis. The temperature range is 2-240 K.

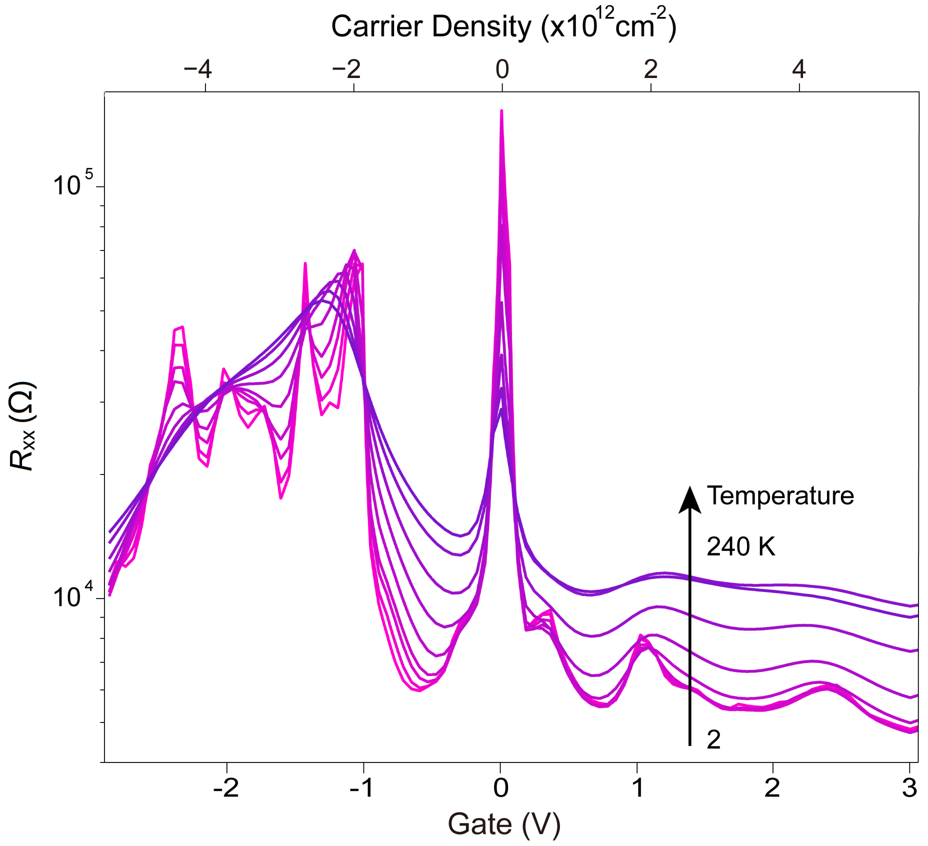

Supplementary Figure 15. **Temperature dependence of the resistance for sample S4.** R_{xx} as a function of carrier density at different temperatures, extracted from Supplementary Figure 12. Notice that this sample has almost identical sets of resistive peaks at low temperature as sample S12. We therefore assume that the alignment is the same as sample S12, so as to the carrier concentrations for each peaks.

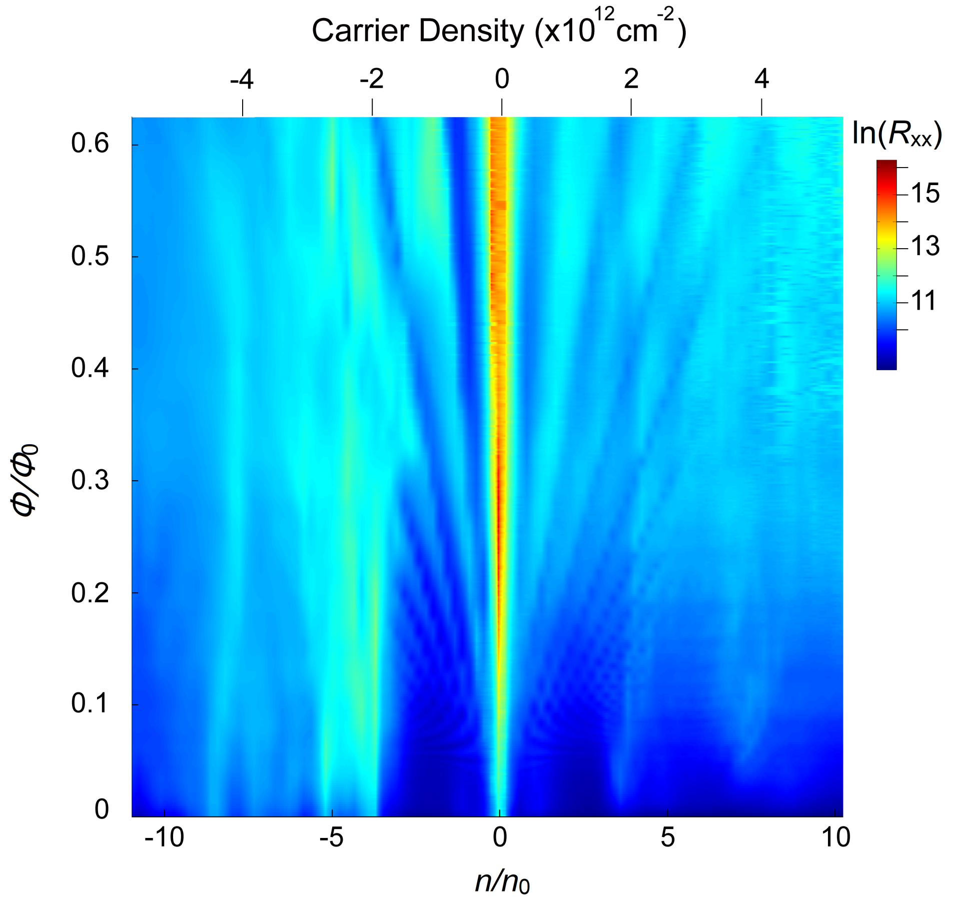

Supplementary Figure 16. **Hofstadter butterfly for sample S4.** Landau fan diagram for device S4 plotted in ϕ/ϕ_0 and n/n_0. Data measured at 2 K.

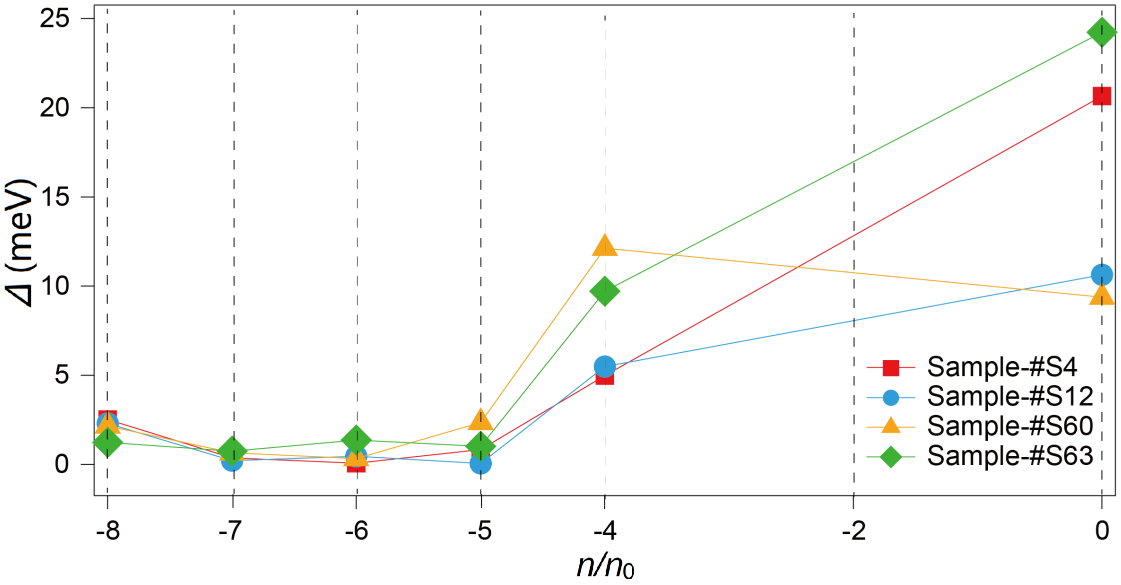

Supplementary Figure 17. **Thermal activation gaps at integer fillings for samples S4, S12, S60, and S63.** It is seen that the thermal activation gaps fitted from the two samples, within the temperature range of 5 to 50 K, are in good agreement.

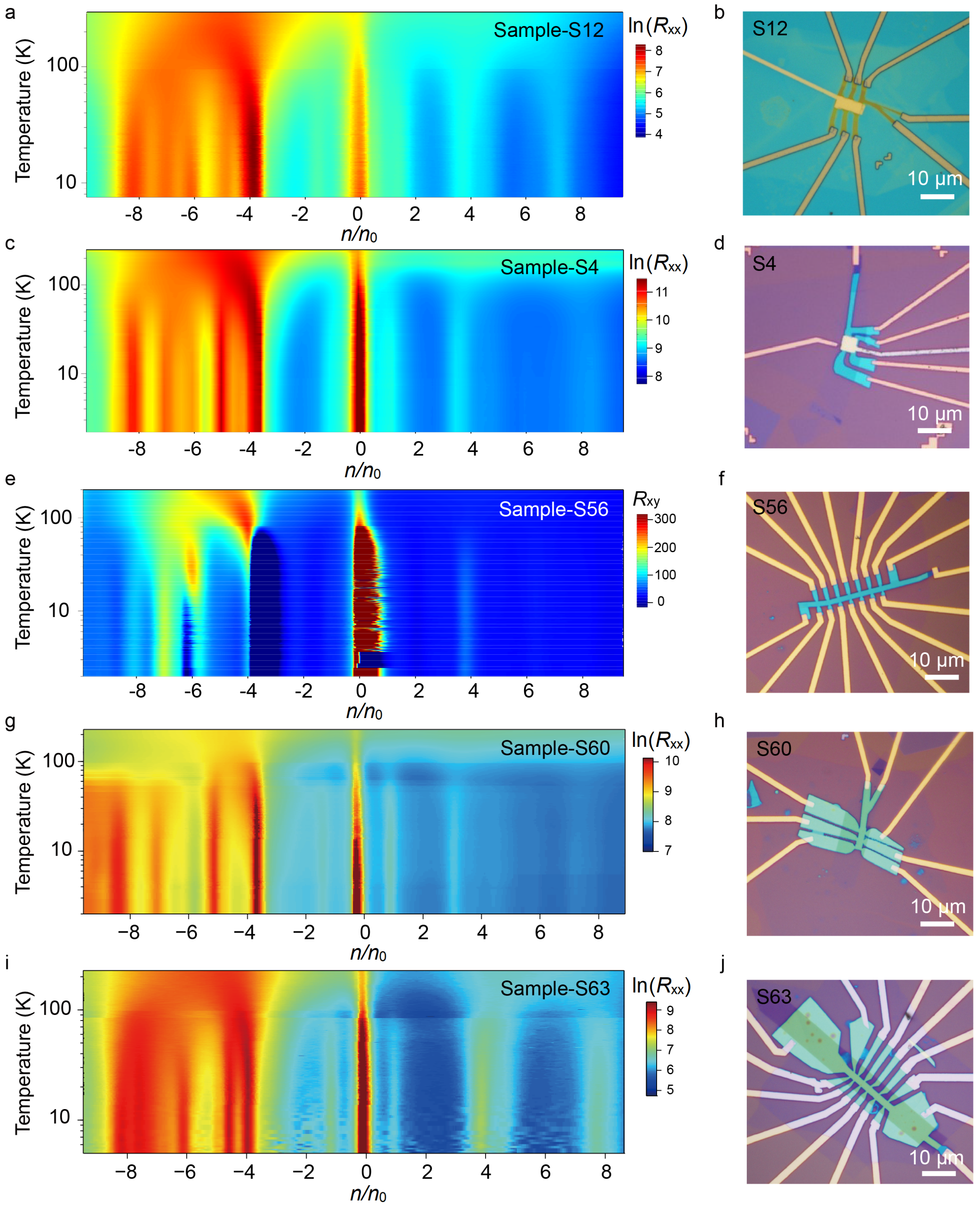

Supplementary Figure 18. **Comparison of field effect curves as a function of temperature for samples S12, S4, S56, S60, and S63.** From top to down are field effect curves as a function of temperature measured in different samples, with the optical images of each corresponding data shown on the right side.

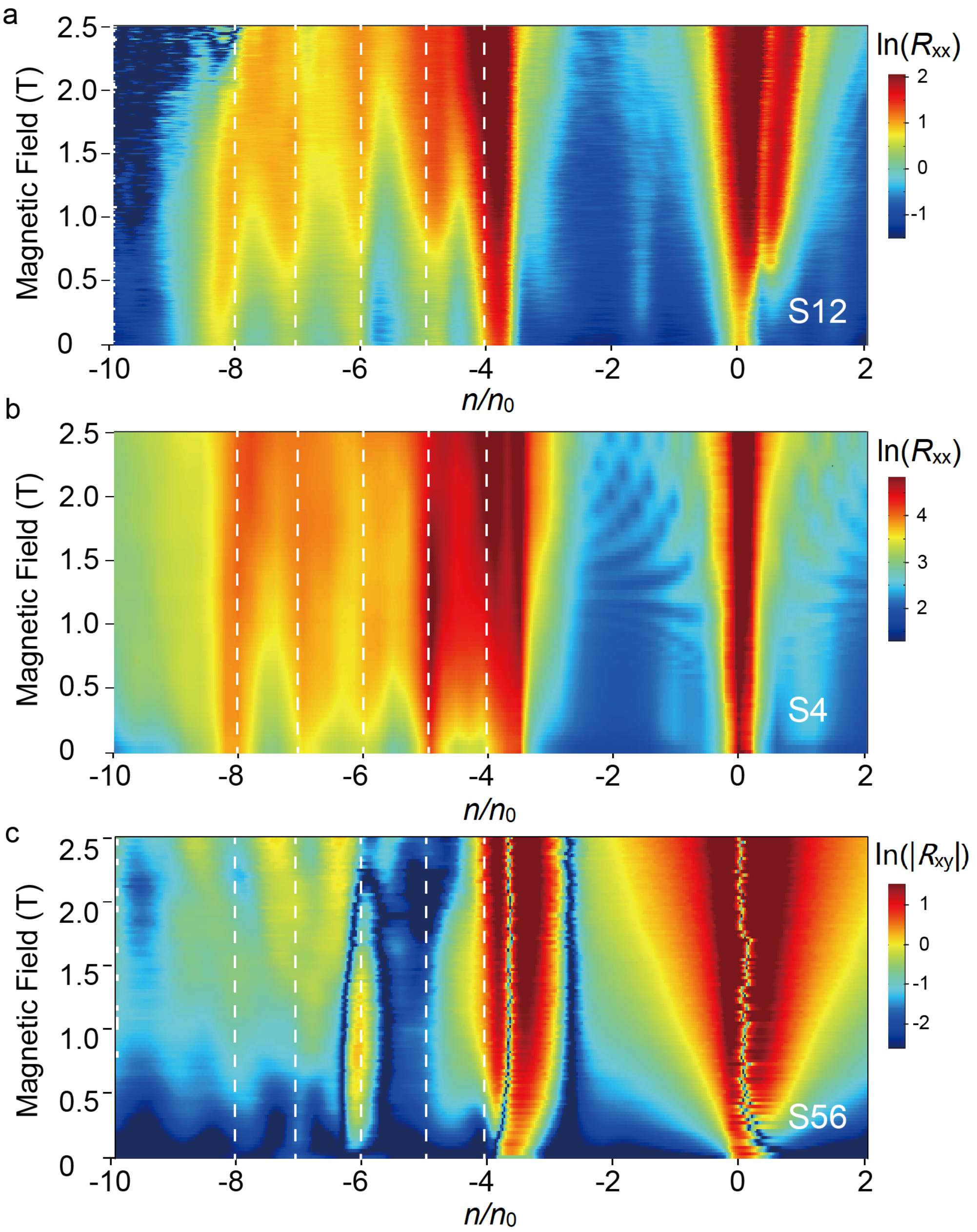

Supplementary Figure 19. **Magneto-transport of different samples in the alignment regime, for samples S4, S12, and S56.** From top to down are magneto-transport recorded in the range of 0 to 2.5 T at 50 mK from S12, S4, and S56, respectively. Notice that sample S56 was measured in its R_{xy} with the absolute value plotted, and the pattern is a bit disturbed due to electron-hole sign switch. Data obtained at 50 mK.

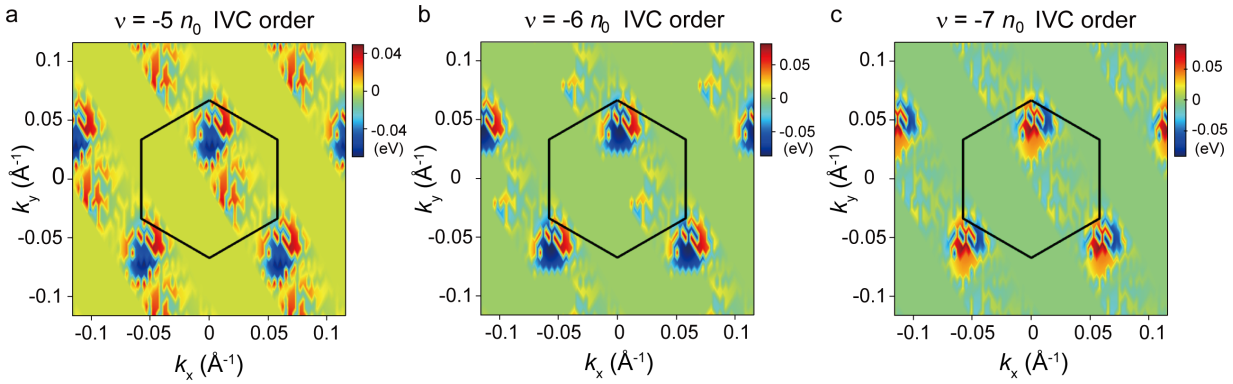

Supplementary Figure 20. **Amplitude of the calculated IVC orders.** (a)-(c) are the distribution of inter-valley coherent (IVC) order in the reciprocal space at different fillings. The average values (by summing the amplitudes at each *k*-point and divided by the mini Brillouin zone) are determined to be 1.42 meV, 1.90 meV and 1.35 meV at the fillings ν=-5, -6, and -7 n_0 respectively. But their maximum value at a particular *k*-point can reach 50-90 meV.

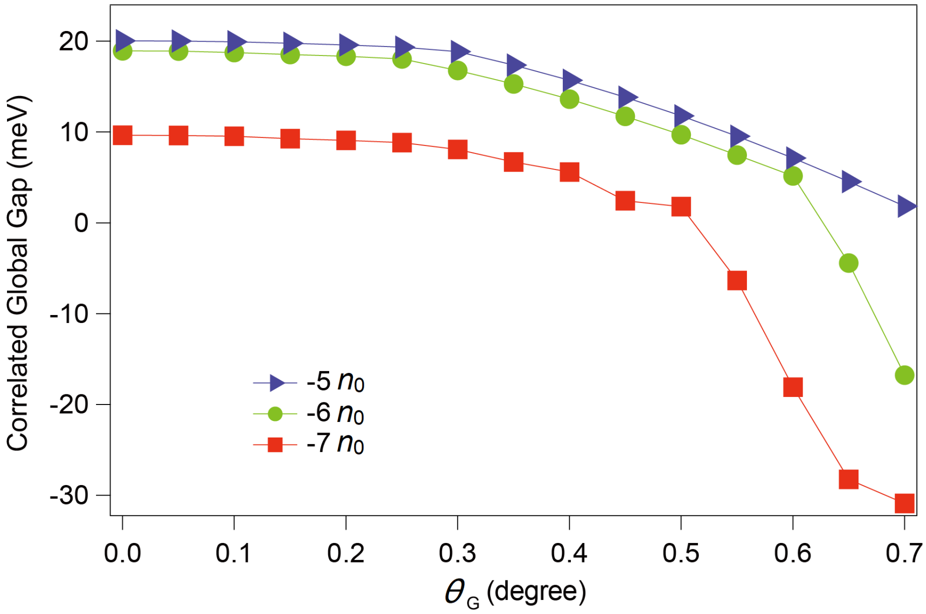

Supplementary Figure 21. **Correlated gaps obtained from Hartree-Fock calculations as a function of θ_G.** The increase of twist angle θ_G (defined in Fig. 2a in the main text) will diminish the correlated gap of each band of -5 n_0, -6 n_0, and -7 n_0. A fixed dielectric constant of $\varepsilon = 4.0$ is used in the calculation.

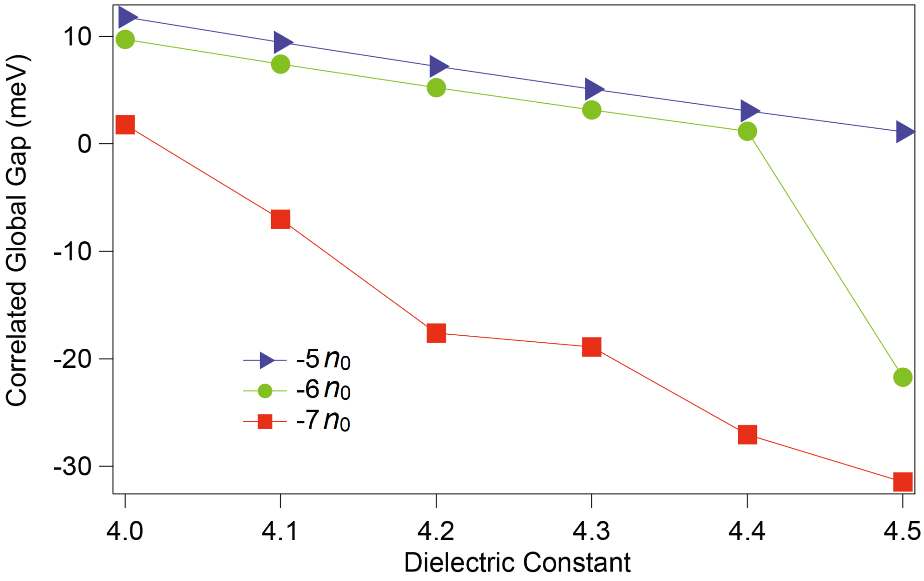

Supplementary Figure 22. **Correlated gaps obtained from Hartree-Fock calculations as a function of dielectric constant.** The increase of dielectric constant will also diminish the correlated gap of each band of -5 n_0, -6 n_0, and -7 n_0. A fixed $\theta_G = 0.5°$ is used in the calculation.

Supplementary Note 1. Continuum model of doubly aligned dual moiré superlattice

In this section, we investigate the simplified case with the top and bottom BN aligned, but rotating the encapsulated graphene with a certain small angle, as depicted in the main text in Fig. 2a. So that the dependences of bandwidths and band gaps with respect to the twist angle of graphene in the middle layer can be calculated, while the top and bottom BN are kept aligned and stay still.

First, we derive the effective continuum Hamiltonian of h-BN/graphene/h-BN from the general tight-binding model. The Hamiltonian of tight-binding model for only p_z orbital can be written as

$$H = -\sum_{i,j} t(\mathbf{R}_i - \mathbf{R}_j)|\mathbf{R}_i\rangle\langle\mathbf{R}_j| + \sum_i V(\mathbf{R}_i)|\mathbf{R}_i\rangle\langle\mathbf{R}_i|, \tag{1}$$

where $\mathbf{R}_i$, $V(\mathbf{R}_i)$ and $t(\mathbf{R}_i - \mathbf{R}_j)$ are the lattice point at i site, the on-site potential at i site and the hopping integral between i and j site, respectively. We assume that the on-site potential of the carbon atom is equal to 0, then we have

$$V_\mathrm{B} = 3.34\,\mathrm{eV}, \quad V_\mathrm{N} = -1.40\,\mathrm{eV}, \tag{2}$$

which are the on-site potential of the boron atom and the nitrogen atoms, respectively [1]. And the hopping integral can be explained by the Slater-Koster-type function of any atomic position [2–4]

$$\begin{aligned} -t(\mathbf{R}) &= V_{pp\pi}\left[1 - \left(\frac{\mathbf{R}\cdot\mathbf{e}_z}{R}\right)^2\right] + V_{pp\sigma}\left(\frac{\mathbf{R}\cdot\mathbf{e}_z}{R}\right)^2 \\ V_{pp\pi} &= V^0_{pp\pi}\mathrm{e}^{\left(-\frac{R-a_0}{r_0}\right)} \\ V_{pp\sigma} &= V^0_{pp\sigma}\mathrm{e}^{\left(-\frac{R-d_0}{r_0}\right)}. \end{aligned} \tag{3}$$

Here, $\mathbf{R}$ and $\mathbf{e}_z$ are the vector distance and the unit vector perpendicular to the layers, respectively. $a_0 = a/\sqrt{3}$ = 0.142 nm is the distance of nearest neighbour A and B atom on graphene and d_0 = 0.335 nm is the interlayer spacing of graphene. $V^0_{pp\pi}$ and $V^0_{pp\sigma}$ is the transfer integral between the nearest neighbor atoms contributed by the π bonds and the σ bonds, respectively. Here, we take $V^0_{pp\pi}$ = -2.7 eV and $V^0_{pp\sigma}$ = 0.48 eV as the reference [5].

We now consider the intralayer interaction and the low-energy effective theory of graphene. The electronic properties of graphene is mainly contributed by electronic states at $\mathbf{K}$ and $\mathbf{K}'$ points [6, 7]. Then the effective Hamiltonian can be derived from the tight-binding model in the low-energy limit. In present case, $\mathbf{K}$ point and $\mathbf{K}'$ point are expressed by $\mathbf{K}_\xi = -\xi(2\mathbf{a}^*_1 + \mathbf{a}^*_2)/3$ where

$\xi = \pm 1$ for $\mathbf{K}$ and $\mathbf{K}'$ points, respectively. The Hamiltonian of the monolayer graphene for intralayer coupling near the $\mathbf{K}_\xi$ points is written as

$$H_\mathrm{G} = -\hbar v \left(\mathbf{k} - \mathbf{K}_\xi\right) \cdot \sigma_\xi, \tag{4}$$

where $\mathbf{k}$ and v is the wave vector and the Fermi velocity of graphene, respectively. The Fermi velocity of graphene is equal to 0.8×10^6 m/s [8]. Note that in our theoretical calculations, the graphene layer can be twisted by an angle θ with respect to the presumingly exactly aligned top and bottom layers, and this twist angle is manifested as a θ dependent Dirac point $\mathbf{K}_\xi(\theta) = \mathbf{K}_\xi + [0, 4\pi\theta/3a]$. And $\sigma_\xi = (\xi\sigma_x, \sigma_y)$ with Pauli matrix σ_x and σ_y describe the different sublattices.

Similar to the graphene layer, the intralayer coupling of the two h-BN layers can be considered only around $\mathbf{K}'_\xi$ points which $\mathbf{K}'_\xi = -\xi(2\mathbf{a}'^*_1 + \mathbf{a}'^*_2)/3$. Due to the electronic structure of the monolayer h-BN has a big energy gap about 5.0 eV, we approximate the effective Hamiltonian by neglecting the dispersion of wave vector $\mathbf{k}$ [9]

$$H_\mathrm{BN} = \begin{pmatrix} V_\mathrm{N} & 0 \\ 0 & V_\mathrm{B} \end{pmatrix}. \tag{5}$$

This is justified when θ is small, because $\mathbf{K}_\xi$ and $\mathbf{K}'_\xi$ are close to each other, and the graphene's electronic states near $\mathbf{K}_\xi$ are coupled only with the h-BN's states near $\mathbf{K}'_\xi$ by the long-range interlayer coupling.

Then we derive the interlayer coupling matrix, and the intralayer coupling Hamiltonian. The Bloch wavefunction can be obtained by the Fourier transformation from the real space basis

$$|\mathbf{k}, X_L\rangle = \frac{1}{\sqrt{N}} \sum_{\mathbf{R}_{X_L}} \mathrm{e}^{i\mathbf{k}\cdot\mathbf{R}_{X_L}} |\mathbf{R}_{X_L}\rangle, \tag{6}$$

where $\mathbf{R}_{X_L}$ is the atom position with different layers and sublattices which $X = \mathrm{A, B}$ denotes different sublattices and $L = 1, 2$ represents different layers. We define the lattice vectors between two atom positions as follows

$$\begin{aligned} \mathbf{R}_X &= n_1\mathbf{a}_1 + n_2\mathbf{a}_2 + \tau_X \\ \mathbf{R}_{X'} &= n_1\mathbf{a}'_1 + n_2\mathbf{a}'_2 + \tau_{X'}, \end{aligned} \tag{7}$$

where τ_X and $\tau_{X'}$ are the lattice vectors between two sublattice atoms A and B in the graphene layer and h-BN layers, respectively. Therefore, the interlayer coupling Hamiltonian in tight-binding model can be expressed by

$$U = -\sum_{X,X'} t_{XX'}(\mathbf{R}_X - \mathbf{R}_{X'})|\mathbf{R}_{X'}\rangle\langle\mathbf{R}_X| + h.c. \tag{8}$$

We substitute the Bloch function into Eq. (8)

$$
\begin{aligned}
U_{X'X}(\mathbf{k}',\mathbf{k}) &= -\frac{1}{\sqrt{N'N}}\sum_{\mathbf{R}_X,\mathbf{R}_{X'}} -t_{X'X}(\mathbf{R}_{X'}-\mathbf{R}_X)\mathrm{e}^{i\mathbf{k}\cdot\mathbf{R}_X-i\mathbf{k}'\cdot\mathbf{R}_{X'}} \\
&= \frac{1}{\sqrt{N'N}}\sum_{\mathbf{R}_X}\mathrm{e}^{i(\mathbf{k}-\mathbf{k}')\cdot\mathbf{R}_X}\sum_{\mathbf{R}_{X'}} t_{X'X}(\mathbf{R}_{X'}-\mathbf{R}_X)\mathrm{e}^{i\mathbf{k}'\cdot(\mathbf{R}_{X'}-\mathbf{R}_X)}.
\end{aligned} \tag{9}
$$

Then we use the in-plane Fourier transformation

$$
t_{X'X}(\mathbf{r}+\mathbf{d}_z) = \frac{1}{\sqrt{N'N}}\int \widetilde{t}_{X'X}(\mathbf{q})\mathrm{e}^{i\mathbf{q}\cdot\mathbf{r}}\mathrm{d}\mathbf{q}, \tag{10}
$$

where $\mathbf{r}+\mathbf{d}_z = (\tau_{X'}-\tau_X)$ and the integral in $\mathbf{q}$ is taken over two-dimensional reciprocal space. The Eq. (9) can be simplified as

$$
U_{X'X}(\mathbf{k}',\mathbf{k}) = \sum_{\mathbf{G},\mathbf{G}',\tau_X,\tau_{X'}} \tilde{t}_{X'X}(\mathbf{k}+\mathbf{G})\mathrm{e}^{-i\mathbf{G}\cdot\tau_X+i\mathbf{G}'\cdot\tau_{X'}}\delta_{\mathbf{k}+\mathbf{G},\mathbf{k}'+\mathbf{G}'}, \tag{11}
$$

where $\mathbf{G}$ and $\mathbf{G}'$ are the reciprocal lattice vectors in two nearest neighbor layers. To simplify Eq. (11), we used the transformation

$$
\begin{aligned}
&\sum_{n_1,n_2}\mathrm{e}^{i(\mathbf{q}-\mathbf{k}')\cdot(n_1\mathbf{a}_1-n_2\mathbf{a}_2)} = N'\sum_{\mathbf{G}'}\delta_{\mathbf{q}-\mathbf{k}',\mathbf{G}'} \\
&\sum_{\mathbf{R}_X}\mathrm{e}^{i(\mathbf{k}-\mathbf{k}'-\mathbf{G}')\cdot\mathbf{R}_X} = N\mathrm{e}^{i(\mathbf{k}-\mathbf{k}'-\mathbf{G}')\cdot\tau_X}\sum_{\mathbf{G}}\delta_{\mathbf{k+G},\mathbf{k}'+\mathbf{G}'}.
\end{aligned} \tag{12}
$$

The hopping integral $\tilde{t}(\mathbf{k})$ decays rapidly as $\mathbf{k}$ increases. We only consider the electronic state near $\mathbf{K}$ or $\mathbf{K}'$ points, the Hamiltonian can be simplified as

$$
U = \begin{pmatrix} U_{\mathrm{A_2A_1}} & U_{\mathrm{A_2B_1}} \\ U_{\mathrm{B_2A_1}} & U_{\mathrm{B_2B_1}} \end{pmatrix} = \tilde{t}(\mathbf{K}_\xi)\left[\begin{pmatrix} 1 & 1 \\ 1 & 1 \end{pmatrix} + \begin{pmatrix} 1 & \omega^{-\xi} \\ \omega^{\xi} & 1 \end{pmatrix}\mathrm{e}^{i\xi\mathbf{G}_1^{\mathrm{M}}\cdot\mathbf{r}} + \begin{pmatrix} 1 & \omega^{\xi} \\ \omega^{-\xi} & 1 \end{pmatrix}\mathrm{e}^{i\xi(\mathbf{G}_1^{\mathrm{M}}+\mathbf{G}_2^{\mathrm{M}})\cdot\mathbf{r}}\right], \tag{13}
$$

where we used $\mathbf{G}_i\cdot\delta(\mathbf{r}) = \mathbf{G}_i^{\mathrm{M}}\cdot\mathbf{r}$ and $\tilde{t}(\mathbf{K}_\xi) = 0.152\,\mathrm{eV}$. The single particle Hamiltonian of h-BN/graphene/h-BN system which the top and bottom layer of h-BN are aligned is

$$
H = \begin{pmatrix} H_{\mathrm{BN}} & U & 0 \\ U^\dagger & H_{\mathrm{G}} & U^\dagger \\ 0 & U & H_{\mathrm{BN}} \end{pmatrix}. \tag{14}
$$

Due to we only consider the low-energy spectrum, we can make H_{BN} as the second-order perturbation of the effective Hamiltonian. The effective Hamiltonian matrix is

$$
H_1 = H_{\mathrm{G}} + 2U^\dagger(-H_{\mathrm{BN}})^{-1}U. \tag{15}
$$

It is noted that the lattice constant of BN is $a_{\rm BN} = 2.504$ Å, which is slightly larger than the graphene's lattice constant $a = 2.46$ Å. It induces isotropic expansion $1+\varepsilon = a_{\rm BN}/a = 1.018$ and makes the moiré lattice constant L become

$$L = \frac{1+\varepsilon}{\sqrt{\varepsilon^2 + 2(1+\varepsilon)(1-\cos\theta)}} a, \tag{16}$$

where ε represents the lattice mismatch and θ refers to the twist effect.

On the other band, when the the top and bottom layer of h-BN have relative twist angle about 60 degree, The single particle Hamiltonian of h-BN/graphene/h-BN system can be written as

$$H = \begin{pmatrix} H_{\rm BN} & U & 0 \\ U^\dagger & H_{\rm G} & U^\dagger \\ 0 & U & H_{\rm BN}^{'} \end{pmatrix}, \tag{17}$$

where

$$H_{\rm BN}' = \begin{pmatrix} V_{\rm B} & 0 \\ 0 & V_{\rm N} \end{pmatrix}. \tag{18}$$

The effective Hamiltonian of Eq. (17) by second-order perturbation theory is

$$H_1 = H_{\rm G} + U^\dagger \left(-H_{\rm BN}\right)^{-1} U + U^\dagger \left(-H_{\rm BN}^{'}\right)^{-1} U. \tag{19}$$

The energy spectrum of different types of h-BN/graphene/h-BN system can be calculated by Eq. (15) and Eq. (19), shown in Fig. 2e in the main text.

Supplementary References

[1] Sławińska, J., Zasada, I. & Klusek, Z. Energy gap tuning in graphene on hexagonal boron nitride bilayer system. *Physical Review B* **81**, 155433 (2010). URL `https://link.aps.org/doi/10.1103/PhysRevB.81.155433`.

[2] Trambly de Laissardire, G., Mayou, D. & Magaud, L. Localization of dirac electrons in rotated graphene bilayers. *Nano Letters* **10**, 804–808 (2010). URL `https://doi.org/10.1021/nl902948m`.

[3] Slater, J. C. & Koster, G. F. Simplified lcao method for the periodic potential problem. *Physical Review* **94**, 1498–1524 (1954). URL `https://link.aps.org/doi/10.1103/PhysRev.94.1498`.

[4] Uryu, S. Electronic states and quantum transport in double-wall carbon nanotubes. *Physical Review B* **69**, 075402 (2004). URL `https://link.aps.org/doi/10.1103/PhysRevB.69.075402`.

[5] Moon, P. & Koshino, M. Electronic properties of graphene/hexagonal-boron-nitride moiré superlattice. *Physical Review B* **90**, 155406 (2014). URL `https://link.aps.org/doi/10.1103/PhysRevB.90.155406`.

[6] DiVincenzo, D. P. & Mele, E. J. Self-consistent effective-mass theory for intralayer screening in graphite intercalation compounds. *Physical Review B* **29**, 1685–1694 (1984). URL `https://link.aps.org/doi/10.1103/PhysRevB.29.1685`.

[7] Semenoff, G. W. Condensed-matter simulation of a three-dimensional anomaly. *Physical Review Letters* **53**, 2449–2452 (1984). URL `https://link.aps.org/doi/10.1103/PhysRevLett.53.2449`.

[8] Moon, P. & Koshino, M. Optical absorption in twisted bilayer graphene. *Physical Review B* **87**, 205404 (2013). URL `https://link.aps.org/doi/10.1103/PhysRevB.87.205404`.

[9] Kindermann, M., Uchoa, B. & Miller, D. L. Zero-energy modes and gate-tunable gap in graphene on hexagonal boron nitride. *Physical Review B* **86**, 115415 (2012). URL `https://link.aps.org/doi/10.1103/PhysRevB.86.115415`.

[10] Wang, Z. *et al.* Composite super-moiré lattices in double-aligned graphene heterostructures. *Science Advances* **5**, eaay8897 (2019).

[11] McGilly, L. J. *et al.* Visualization of moiré superlattices. *Nature Nanotechnology* **15**, 580 (2020).
